# Registration of Stillbirth After Early Fetal Death: A National Population‐Based Analysis

**DOI:** 10.1111/1471-0528.70133

**Published:** 2026-01-06

**Authors:** Ruth J. Matthews, K. Sruthi Vydyula, Ian D. Gallimore, Bradley N. Manktelow, Lucy K. Smith

**Affiliations:** ^1^ Division of Nursing Sciences, Pregnancy and Child Health, School of Healthcare, College of Life Sciences University of Leicester Leicester UK; ^2^ Leicester Medical School, College of Life Sciences University of Leicester Leicester UK

**Keywords:** epidemiology, perinatal, stillbirth

## 1 Introduction

Stillbirth rates are a vital indicator of the quality of care in pregnancy and childbirth and of woman's health [1]. Accurate information on stillbirths is required for monitoring purposes, often utilising civil registrations of stillbirths. However, there are significant methodological challenges in ensuring robust stillbirth statistics, which are essential for accurate surveillance, policy development, and international comparisons [2]. Recommendations have been developed to overcome challenges relating to the inclusion of deaths following termination of pregnancy [3] and inconsistencies in the determination of signs of life [4].

In England and Wales, we have identified an additional challenge arising from the use of stillbirth registration data provided by the Office for National Statistics (ONS) for monitoring mortality. This relates to scenarios where the birth occurs after the registration threshold of 24 weeks’ gestation, but fetal death occurred in utero before this time. The Royal College of Obstetricians and Gynaecologists developed Good Practice Paper No. 4 [5] specifically recommending that a death should not be registered as a stillbirth where fetal death occurred in utero before 24 weeks' gestation, regardless of gestational age at birth. However, anecdotal evidence has suggested that this guidance is not routinely followed in practice, which could lead to inconsistencies in official statistics, inequities for parents, and misclassification of perinatal deaths. This issue is of particular concern in the case of multiple births, where the interval between fetal death and birth is often more prolonged, with birth occurring when one or more surviving babies are born.

To explore these potential registration inconsistencies and examine their impact on stillbirth registration practice, we utilised national surveillance data from MBRRACE‐UK (Mothers and Babies: Reducing Risk through Audits and Confidential Enquiries across the UK) [6], which includes information on both the gestation when the in utero death was confirmed and the gestation at birth. Here, we report the consistency in the implementation of RCOG GPP No. 4 regarding registration of deaths where in utero death occurs before 24 weeks, but birth occurs after the 24‐week stillbirth registration threshold.

## 2 Exploring Inconsistencies in Registration

MBRRACE‐UK conduct national surveillance of perinatal deaths in the UK to monitor perinatal mortality rates. Deaths are reported by individual trusts and health boards, and these reported perinatal deaths are matched with stillbirth and neonatal death registrations from statutory sources across the devolved nations of the UK to ensure complete ascertainment of registered deaths (e.g., ONS for births in England and Wales) [7]. In this analysis, we used data on perinatal deaths to mothers resident in England and Wales who gave birth between 1 January 2013 and 31 December 2023. The dataset included information on gestation at which death was confirmed in utero, gestation at birth, multiplicity of pregnancy and whether the death was registered as a stillbirth with ONS. Our analyses focused on antepartum deaths, i.e., excluding deaths where the baby was alive at the onset of labour or the birthing process but died before birth (intrapartum in utero deaths) or the baby was live born but died after birth (neonatal deaths). We explored the percentage of all reported antepartum deaths where death occurred in utero before 24 weeks’ gestation out of all reported antepartum deaths by multiplicity of birth. We then explored the percentage of in utero deaths occurring before 24 weeks by the gestation at which they were born for both singleton and multiple births, and assessed the percentage of these deaths that had been registered as a stillbirth with ONS. To look at the impact this registration practice had on total registered stillbirths, we calculated the number of registered stillbirths where the death was confirmed before 24 weeks as a percentage of all registered stillbirths.

Of the 23 332 total antepartum deaths for births from 24 weeks’ gestation reported to MBRRACE‐UK in England and Wales between 2013 and 2023, we identified 577 (2.5%) antepartum deaths where the death was confirmed to have occurred in utero before 24 weeks (Table 1). For multiple births, this percentage was much higher at 14% of deaths (223/1 595).

**TABLE 1:** Number of antepartum stillbirths at gestation 24 weeks plus in England and Wales between 1 January 2013 and 31 December 2013, and the number where death was confirmed in utero before 24 weeks, by multiplicity and gestation at birth.

	Number of antepartum stillbirths	Number (%) where death confirmed in utero before 24 weeks
**ALL**	23 332	577 (2.5)
**Multiplicity**		
Singletons	21 737	354 (1.6)
Twins and higher order	1 595	223 (14.0)
**Gestation**		
24 to 26 weeks	4 245	425 (10.0)
27 to 31 weeks	5 377	63 (1.2)
32 to 36 weeks	6 239	53 (0.85)
37+ weeks	7 471	36 (0.48)

The majority of antepartum deaths in singleton pregnancies had death confirmed at 21 to 23 weeks’ gestation (344/354, 97.2%). In multiple pregnancies, there were more deaths confirmed at earlier gestations, with 66.8% between 21 and 23 weeks (149/223), 25% at 18 to 20 weeks (54/223), and 9% (20/223) before 18 weeks’ gestation.

For singleton babies who died before 24 weeks, over 99% (353/354) were born between 24 and 26 weeks’ gestation, with only one of the 354 deaths born after this (at 31 weeks). In contrast for multiples, the interval between death and birth was greater, with only 32% (72/223) of these births occurring at 24 to 26 weeks, 28% (63/223) between 27 and 31 weeks, 23% (52/223) at 32 to 36 weeks and 16% (36/223) at 37 weeks and over.

Table 2 shows that around half of all these deaths (47.8%, 276/577) were legally registered as stillbirths. The percentage was highest for births at 24 to 26 weeks (54.1%, 230/425) and lowest at 32 to 36 weeks (17%, 9/53). Overall, a higher proportion of deaths of singleton babies were registered as stillbirths (54.5%, 193/354) compared to deaths of twins and higher order births (37.2%, 83/223). The rates of registration were very similar for both groups at 24–26 weeks, but a lower overall rate among multiples reflected the lower registration rates among multiples at later gestations.

**TABLE 2:** In utero deaths occurring before 24 weeks where birth/delivery occurred at or after 24 weeks (2013–2023): Numbers, registered stillbirths, and registration percentages (95% confidence interval) by gestational age and multiplicity.

Gestation at birth (weeks)	Multiplicity of birth								
	All births			Singletons			Twins and higher order		
	Total deaths	Registered as stillbirths	% of deaths registered (95% confidence interval)	Total deaths	Registered as stillbirths	% of deaths registered (95% confidence interval)	Total deaths	Registered as stillbirths	% of deaths registered (95% confidence interval)
**24 to 26**	425	230	54.1 (49.3, 58.9)	353	193	54.7 (49.4, 59.9)	72	37	51.4 (39.8, 63.0)
**27 to 31**	63	26	41.3 (29.1, 53.5)	0	0		63	26	41.3 (29.1, 53.5)
**32 to 36**	53	9	17.0 (6.8, 27.1)	1	0	0	52	9	17.3 (7.0, 27.6)
**37+**	36	11	30.6 (15.5, 45.7)	0	0		36	11	30.6 (15.5, 45.7)
**ALL**	577	276	47.8 (43.7, 52.0)	354	193	54.5 (49.3, 59.8)	223	83	37.2 (30.8,43.6)

When examining the number of registered stillbirths where the death was confirmed before 24 weeks as a percentage of all registered stillbirths, we found an additional 5% (83/1 595) of multiple births were registered as a stillbirth compared with only 0.9% (193/21 737) of singleton births.

## 3 Implications of Registration Inconsistency

Our findings reveal that in England and Wales, approximately half of births where death in utero occurred before 24 weeks' gestation but the babies were born after 24 weeks were registered as stillbirths. This is inconsistent with RCOG GPP No. 4 national recommendations [5].

These registration inconsistencies have significant implications for national stillbirth registration data, particularly for multiple births, where nearly 5% of these deaths were registered contrary to the RCOG GPP No. 4 recommendations. This reflects the longer gap between in utero death and birth, with the pregnancy continuing until the birth of the surviving twin or higher‐order birth.

This variation in practice may reflect a lack of awareness of the guidance or suggest that healthcare professionals are using pragmatic, compassionate judgement rather than strict adherence to clinical guidelines. The GPP No. 4 [5] offers one interpretation of pregnancy ending at the time of in utero death. However, some healthcare professionals may interpret pregnancy to end at birth and register accordingly. The provision of a stillbirth certificate and access to parental leave represent tangible acknowledgement of bereavement as well as practical and financial support during recovery for parents [8, 9]. Consequently, in more uncertain scenarios, some healthcare professionals may discuss with parents their preferences for registration, recognising the importance of formal recognition of baby loss for many parents who have experienced the death of their baby.

This tension between following guidance and parent‐centred care highlights a fundamental gap in current policy frameworks. While clinical guidelines appropriately aim to standardise medical definitions for legislative and epidemiological purposes, they may not adequately address the psychological and social needs of bereaved parents. Healthcare professionals appear to bridge this gap through discretionary registration practices that prioritise parental welfare over consistency of registration practices. Since 2024, alternative formal approaches have been developed to offer certification for pregnancy losses before 24 weeks’ of gestation in England [10]. However, they do not facilitate access to other benefits, such as parental leave and pay, for many of these parents, particularly those of singleton babies.

The systematic bias in stillbirth rates through the use of inconsistent application of guidance undermines the accuracy of public health monitoring and international comparisons. This may lead to misallocation of resources, inappropriate policy responses, and difficulties in evaluating the effectiveness of interventions aimed at reducing stillbirth rates. Furthermore, the disproportionate effect on multiple births may create artificial disparities in reported stillbirth rates between singleton and multiple pregnancies, potentially masking genuine risk factors or improvements in care. This statistical bias could impact clinical research, guideline development, and resource allocation for multiple pregnancy management.

Our findings demonstrate the need to distinguish between registration data and dedicated surveillance data for public health monitoring purposes. While registration systems serve important legal and administrative functions, their susceptibility to compassionate decision‐making renders them unreliable for epidemiological surveillance. The identification of systematic bias in approximately half of the relevant cases highlights fundamental limitations in using registration data as the primary source for stillbirth monitoring. Surveillance systems such as MBRRACE‐UK, which collect data specifically for epidemiological purposes with standardised definitions and clinical validation, offer high‐quality data for public health monitoring. These systems can maintain strict adherence to clinical definitions, while registration systems focus on the requirements of civil registration and also families' needs for recognition and support.

## 4 Data Limitations

This study is limited by its focus on registration data and cannot definitively establish the motivations behind healthcare professionals' decisions. While our interpretation suggests compassionate intentions, other factors such as uncertainty about gestational age determination or misunderstanding of guidelines may also contribute to inconsistent practices. Further qualitative research exploring healthcare professionals' perspectives would strengthen the understanding of this phenomenon.

Data considerations include small numbers and reliability of reporting, given that this is performed by healthcare professionals, often in stressful situations. Specifically, MBRRACE‐UK data does not include the mode of confirmation of the date of death, whether this is via ultrasound scan, post‐mortem examination, or a professional opinion, which could alter the reliability of the data. Other explanations may be increased certainty around the interval between date of death and date of birth at higher gestations in multiple pregnancies, where increased surveillance, such as ultrasound scanning, is undertaken.

## 5 Conclusion

Current frameworks create an artificial binary where parents only receive full recognition through stillbirth registration, providing access to parental leave and official legal registration. This may motivate healthcare professionals to circumvent guidelines in the interests of compassionate care. This is leading to biased registration data and impacts the monitoring of stillbirth outcomes. GPP No. 4 is currently under revision, and we hope this will improve consistency. Alternative surveillance data are necessary to ensure robust public health monitoring, and policymakers should reflect on how to address inequalities in access to parental leave and bereavement support to remove the practical incentives that may be driving inconsistent registration practices.

## Author Contributions

L.K.S. had the original idea, which was then developed in discussion with R.J.M., K.S.V., B.N.M. and I.D.G. R.J.M. analysed the data, and all authors contributed to the interpretation of the data. L.K.S., R.J.M. and K.S.V. wrote the first draft of the manuscript, which was edited by I.D.G. and B.N.M. All authors approved the final manuscript.

## Funding

The MBRRACE‐UK collaboration is commissioned by the Healthcare Quality Improvement Partnership (HQIP) to deliver the Maternal, Newborn and Infant Clinical Outcome Review Programme on behalf of NHS England, the devolved Governments of Wales, Scotland, and Northern Ireland, and the Governments of the British Crown Dependencies. Lucy Smith (Advanced Fellowship, NIHR301421) is also funded by the NIHR for this project.

## Conflicts of Interest

Completed disclosure of interests forms are available to view online as supporting information. The authors have no conflicts of interest; no financial relationships with any organisations that might have an interest in the submitted work in the previous three years; no other relationships or activities that could appear to have influenced the submitted work. L.K.S. received a personal award from the NIHR (Advanced Fellowship, NIHR301421). R.J.M., L.K.S., B.N.M. and I.D.G. are part of the MBRRACE‐UK (Mothers and Babies: Reducing Risk through Audits and Confidential Enquiries across the UK) collaboration, funded by HQIP. The views expressed are those of the authors and not necessarily those of the NIHR or the Department of Health and Social Care.

## Ethics Statement

Data held by MBRRACE‐UK (Mothers and Babies: Reducing Risk through Audits and Confidential Enquiries across the UK) were collected in England and Wales without consent with approval of the secretary of state for health and social care under section 251 of the NHS Act 2006 (15/CAG/0119). The legal basis for this activity is Article 6 (1)(e) and Article 9 (2)(i) under the General Data Protection Regulation.

## Data Availability

The data that support the findings of this study are available from Healthcare Quality Improvement Partnership. Restrictions apply to the availability of these data, which were used under license for this study. Data are available from the author(s) with the permission of Healthcare Quality Improvement Partnership.

## References

[1] V. Flenady , A. M. Wojcieszek , P. Middleton , et al., “Stillbirths: Recall to Action in High‐Income Countries,” Lancet 387, no. 10019 (2016): 691–702. 26794070 10.1016/S0140-6736(15)01020-X

[2] M. Gissler , A. D. Mohangoo , B. Blondel , J. Chalmers , A. Macfarlane , and A. Gaizauskiene , “Perinatal Health Monitoring in Europe: Results From the EURO‐PERISTAT Project,” Informatics for Health and Social Care 35 (2010): 64–79. 20726736 10.3109/17538157.2010.492923

[3] B. Blondel , M. Cuttini , A. D. Hindori‐Mohangoo , et al., “How Do Late Terminations of Pregnancy Affect Comparisons of Stillbirth Rates in Europe? Analyses of Aggregated Routine Data From the Euro‐Peristat Project,” BJOG: An International Journal of Obstetrics and Gynaecology 125, no. 2 (2018): 226–234. 28557289 10.1111/1471-0528.14767

[4] L. K. Smith , B. Blondel , and J. Zeitlin , “Producing Valid Statistics When Legislation, Culture and Medical Practices Differ for Births at or Before the Threshold of Survival: Report of a European Workshop,” BJOG: An International Journal of Obstetrics & Gynaecology 127, no. 3 (2020): 314–318. 31580509 10.1111/1471-0528.15971 PMC7003918

[5] Royal College of Obstetricians & Gynaecologists (RCOG) , “Registration of stillbirths and certification for pregnancy loss before 24 weeks of gestation. Good Practice No. 4,” (2005), https://www.rcog.org.uk/media/030jxnk5/goodpractice4registrationstillbirth2005.pdf. 15984641

[6] I. D. Gallimore , R. J. Matthews , G. L. Page , et al., “MBRRACE‐UK Perinatal Mortality Surveillance, UK Perinatal Deaths of Babies Born in 2023: State of the Nation Report,” TIMMS (Department of Population Health Sciences, University of Leicester, 2025).

[7] E. Draper , I. Gallimore , L. Smith , et al., “MBRRACE‐UK Perinatal Mortality Surveillance: Technical Manual,” in The Infant Mortality and Morbidity Studies (Department of Population Health Sciences, University of Leicester, 2023).

[8] Z. Clark‐Coates and S. Collinge , The Independent Pregnancy Loss Review: Care and Support When Baby Loss Occurs Before 24 Weeks Gestation (His Majesty’s Stationery Office, 2023).

[9] L. K. Smith , J. Dickens , R. Bender Atik , C. Bevan , J. Fisher , and L. Hinton , “Parents' Experiences of Care Following the Loss of a Baby at the Margins Between Miscarriage, Stillbirth and Neonatal Death: A UK Qualitative Study,” BJOG: An International Journal of Obstetrics and Gynaecology 127, no. 7 (2020): 868–874. 31976622 10.1111/1471-0528.16113 PMC7383869

[10] Department of Health and Social Care , “Baby loss certificate launched to recognise parents’ grief [press release],” (2024), https://www.gov.uk/government/news/baby‐loss‐certificate‐launchedto‐recognise‐parents‐grief.

